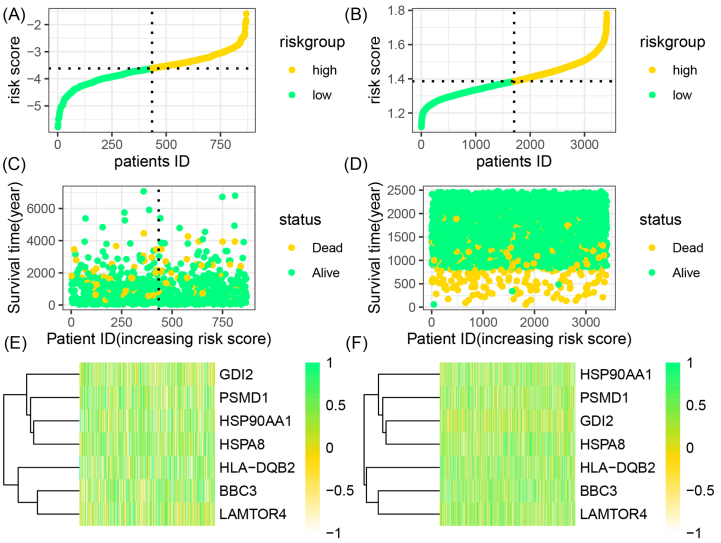# Molecular subtype identification and prognosis stratification based on lysosome-related genes in breast cancer

**DOI:** 10.1016/j.heliyon.2024.e25643

**Published:** 2024-02-07

**Authors:** Xiaozhen Liu, Kewang Sun, Hongjian Yang, Dehomg Zou, Lingli Xia, Kefeng Lu, Xuli Meng, Yongfeng Li

**Affiliations:** a General Surgery, Cancer Center, Department of Breast Surgery, Zhejiang Provincial People’s Hospital (Affiliated People’s Hospital, Hangzhou Medical College), Hangzhou, Zhejiang, 310014, China; b Department of Breast Surgery, Institute of Cancer Research and Basic Medical Sciences of Chinese Academy of Sciences (Zhejiang Cancer Hospital), Hangzhou, Zhejiang, 310022, China; c Department of Outpatient Service, Institute of Cancer Research and Basic Medical Sciences of Chinese Academy of Sciences (Zhejiang Cancer Hospital), Hangzhou, Zhejiang, 310022, China; d Cancer Center, Department of Ultrasound Medicine, Zhejiang Provincial People’s Hospital (Affiliated People’s Hospital, Hangzhou Medical College), Hangzhou, Zhejiang, 310014, China

**Keywords:** Breast cancer, Lysosomes, Prognosis, Consensus clustering, Nomogram

## Abstract

**Background:**

Lysosomes are known to have a significant impact on the development and recurrence of breast cancer. However, the association between lysosome-related genes (LRGs) and breast cancer remains unclear. This study aims to explore the potential role of LRGs in predicting the prognosis and treatment response of breast cancer.

**Methods:**

Breast cancer gene expression profile data and clinical information were downloaded from TCGA and GEO databases, and prognosis-related LRGs were screened for consensus clustering analysis. Lasso Cox regression analysis was used to construct risk features derived from LRGs, and immune cell infiltration, immune therapy response, drug sensitivity, and clinical pathological feature differences were evaluated for different molecular subtypes and risk groups. A nomogram based on risk features derived from LRGs was constructed and evaluated.

**Results:**

Our study identified 176 differentially expressed LRGs that are associated with breast cancer prognosis. Based on these genes, we divided breast cancer into two molecular subtypes with significant prognostic differences. We also found significant differences in immune cell infiltration between these subtypes. Furthermore, we constructed a prognostic risk model consisting of 7 LRGs, which effectively divides breast cancer patients into high-risk and low-risk groups. Patients in the low-risk group have better prognostic characteristics, respond better to immunotherapy, and have lower sensitivity to chemotherapy drugs, indicating that the low-risk group is more likely to benefit from immunotherapy and chemotherapy. Additionally, the risk score based on LRGs is significantly correlated with immune cell infiltration, including CD8 T cells and macrophages. This risk score model, along with age, chemotherapy, clinical stage, and N stage, is an independent prognostic factor for breast cancer. Finally, the nomogram composed of these factors has excellent performance in predicting overall survival of breast cancer.

**Conclusions:**

In conclusion, this study has constructed a novel LRG-derived breast cancer risk feature, which performs well in prognostic prediction when combined with clinical pathological features.

## 1 Introduction

Breast cancer is a prevalent malignant tumor among women worldwide, and its incidence continues to increase [1,2]. Breast cancer is classified into multiple subtypes, among which triple-negative breast cancer has a poor prognosis and lacks effective treatment targets [3]. The prognosis and treatment of breast cancer mainly depend on its stage and subtype. Currently, although there are multiple molecular markers for predicting breast cancer prognosis, such as Oncotype DX and MammaPrint [4], there is still a lack of reliable single biomarker to detect breast cancer prognosis and predict the efficacy of drug treatment. Therefore, simpler models based on new phenotypes still need to be developed. Although significant progress has been made in patient treatment, breast cancer still has a certain risk of recurrence, and further research is needed to identify genes responsible for long-term survival, develop new treatment methods, or discover new biomarkers.

Lysosomes are cellular organelles responsible for the degradation and recycling of cellular materials. They play a critical role in various cellular processes, including cell death, immune response, energy metabolism, cell signaling, and receptor endocytosis [5,6]. Abnormal lysosomal function may lead to the accumulation of cellular waste and inhibition of cell death, thereby promoting tumor growth and metastasis [7]. Lysosomes also participate in some cancer-related signaling pathways, such as autophagy and apoptosis [8,9]. Recent studies have found that some lysosomal-related mechanisms play a key role in the development of breast cancer and are closely related to drug resistance and prognosis in prostate cancer [10,11]. Although lysosomal-related genes (LRGs) may be promising biomarkers for predicting cancer prognosis [12,13], few studies have focused on establishing LRG-based prognostic models for breast cancer.

As mentioned above, lysosomal-related pathways are crucial in breast cancer. In this study, we screened lysosomal-related genes associated with breast cancer prognosis and established a novel lysosomal-related feature combined with clinical pathological features to predict the prognosis of breast cancer patients. This may provide a powerful treatment option and prediction tool for breast cancer.

## 2 Materials and methods

### 2.1 Data collection and processing

The transcriptional data and corresponding clinical pathological information of the TCGA-BRCA project were downloaded from the TCGA database. Cases with incomplete clinical pathological information and a prognosis time of less than 30 days were excluded. The validation dataset GSE96058 was obtained from the GEO database, which includes information on 3406 cases.

### 2.2 Molecular subtyping

A total of 876 LRGs were obtained from the Gene Ontology (GO) database (http://geneontology.org/). The limma package was utilized to analyze the differential expression of these LRGs in breast cancer, and differentially expressed LRGs (deLRGs) were selected with an adjusted p-value <0.05 as the threshold. Prognostic-related deLRGs were identified using univariate Cox regression analysis, and deLRGs with p < 0.05 were considered to have significant prognostic relevance. Finally, the expression matrix of prognostic-related deLRGs was subjected to Consensus clustering analysis using the ConsensusClusterPlus R package.

### 2.3 Construction and evaluation of prognostic models

The deLRGs that are related to prognostics were compressed further through Lasso Cox regression analysis. Subsequently, a risk model was created using the formula: riskscore = ∑(βi*coefi) (‘i’ = the number of prognostic LRGs, 'βi' represents the expression of each LRGs, ‘coefi’ represents the coefficient of each LRGs. The training and validation sets were segregated into high-risk and low-risk groups based on the median value of the risk score. Survival analysis was then employed to assess the prognostic differences between the high-risk and low-risk groups. The performance of the prognostic risk model in predicting overall survival for 1, 3, and 5 years was evaluated using receiver operating characteristic (ROC) curves.

### 2.4 Gene set enrichment analysis

In order to investigate the signaling pathways between various risk groups, the “clusterProfiler” package in R was utilized for gene set enrichment analysis. The enriched signaling pathways were then selected based on a P-value <0.05, and a bubble chart was generated to visualize the results.

### 2.5 Immune infiltration analysis

The CIBERSORT R package was employed to conduct immune cell infiltration analysis, which assesses the infiltration of 22 immune cells using transcriptomic data. Furthermore, the 22 immune cells can be categorized into four types for analysis, namely dendritic cells, lymphocytes, macrophages, and mast cells. The immune infiltration was compared between different molecular subtypes and risk groups in the TCGA-BRCA cohort.

### 2.6 Analysis of immune therapy response

The evaluation of immune therapy response can be assessed through two methods, namely Tumor Immune Dysfunction and Exclusion (TIDE) and Immunophenoscore (IPS). TIDE analysis is conducted using a website tool (http://tide.dfci.harvard.edu) and standardized transcriptome data as input, which calculates scores such as TIDE, CAF, Merck18, Dysfunction, and Exclusion. On the other hand, IPS analysis is computed using The Cancer Immunome Atlas (TICA), with TCGA database case IDs as input, to obtain IPS scores between different risk groups in four different scenarios: CTLA-PD1-, CTLA+PD1-, CTLA-PD1+, and CTLA+PD1+.

### 2.7 Chemotherapy drug sensitivity analysis

The evaluation of drug sensitivity is conducted using the pRRophetic R package, which assesses the sensitivity of the most frequently used 9 drugs in the TCGA-BRCA cohort's drug treatment data. The differences in drug sensitivity between different risk groups are then compared.

### 2.8 Construction and evaluation of nomograms

The analysis of independent prognostic factors for breast cancer is conducted through univariate Cox and multivariate Cox regression analysis. Subsequently, a nomogram is constructed using the independent prognostic factors. The rms R package is utilized for nomogram analysis and construction, while calibration curves and decision curves are employed to evaluate the predictive performance of the nomogram.

### 2.9 Statistical analysis

The data analysis and visualization are conducted using R (version 4.2.2), with the Wilcoxon test utilized for difference analysis between two groups. Additionally, Kaplan-Meier (KM) survival curves and log-rank tests are employed for survival analysis. A P value of less than 0.05 is considered statistically significant.

## 3 Results

### 3.1 Prognostic lysosome-related genes and their molecular subtypes

**Fig. 1:**
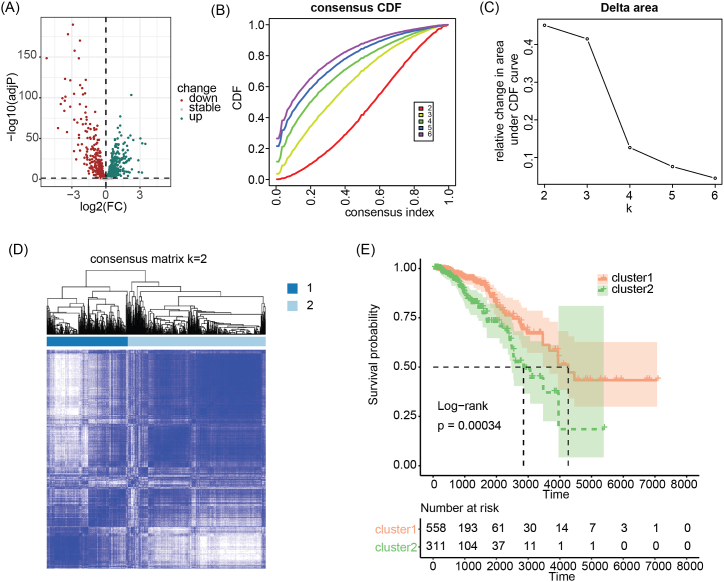
Differential expression and prognosis analysis of lysosome-related genes and molecular subtyping of breast cancer. (A) Differential expression of lysosome-related genes in breast cancer. (B–D) Consensus clustering based on prognosis-related lysosome-related genes. (E) Survival analysis shows significant prognostic differences between two breast cancer molecular subtypes derived from lysosome-related genes.

Fig. 1A displays the differential expression of lysosome-related genes, with 389 genes upregulated and 320 genes downregulated. Prognostic analysis revealed that 176 of these genes are significantly associated with breast cancer prognosis. Moreover, we utilized these prognostic-related deLRGs to molecularly subtype the TCGA-BRCA cohort. The results demonstrated that when k = 2, a good consistency grouping can be achieved (Fig. 1B–D). Survival analysis indicated a significant prognostic difference between the two molecular subtypes (p = 0.00034), with cluster 1 exhibiting a significantly better prognosis than cluster 2 (Fig. 1E).

### 3.2 Risk model of lysosome-related genes

**Fig. 2:**
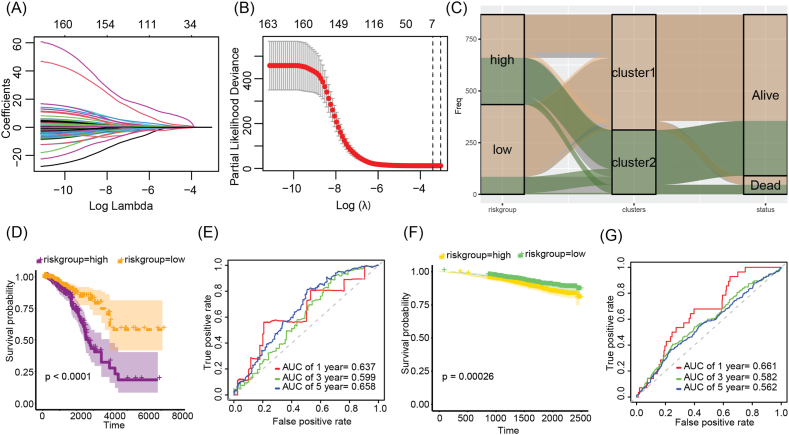
Construction and evaluation of breast cancer prognosis model based on lysosome-related genes. (A–B) Lasso Cox regression analysis results in a 7-genes risk signature. (C) Sankey diagram of the two clusters, two risk groups, and two clinical outcomes. (D) Survival analysis shows that low-risk group patients in the TCGA-BRCA cohort have significantly better prognosis than high-risk group patients. (E) ROC curve shows that the risk model derived from lysosome-related genes significantly predicts the AUC values of 1, 3, and 5-year overall survival of breast cancer in the TCGA-BRCA cohort, which are 0.637, 0.599, and 0.658, respectively. (F) Survival analysis shows that low-risk patients in the GSE96058 cohort have significantly better prognosis than high-risk patients. (G) ROC curve shows that the risk model derived from lysosome-related genes significantly predicts the AUC values of 1, 3, and 5-year overall survival of breast cancer in the GSE96058 cohort, which are 0.661, 0.582, and 0.562, respectively.

To construct a molecular model derived from LRGs, Lasso Cox regression analysis was conducted based on the previous univariate Cox regression analysis (Fig. 2A and B). Ultimately, 7 genes were included in the risk model, and the calculation formula for the risk score is as follows: riskscore = −0.01767817 * BBC3 + 0.00861022 * GDI2 − 0.03530289 * HLA-DQB2 + 0.10365645 * HSP90AA1 + 0.07642630 * HSPA8 − 0.03133462 * LAMTOR4 + 0.04636296 * PSMD1. The case grouping, survival outcome, and related gene expression in the training set and validation set are shown in Supplementary Fig. S1. The distribution and relationship of two clusters, two risk groups, and two clinical outcomes was illustrated in Fig. 2C. Survival analysis showed that in the TCGA-BRCA cohort, patients in the low-risk group had significantly better prognosis than those in the high-risk group (Fig. 2D, p < 0.0001). The AUC values of this risk feature in predicting the overall survival of TCGA-BRCA cohort at 1, 3, and 5 years were 0.637, 0.599, and 0.658, respectively (Fig. 2E). In the GSE96058 cohort, patients in the low-risk group also had significantly better prognosis than those in the high-risk group (Fig. 2F, p = 0.00026), and the AUC values of this model in predicting the overall survival of GSE96058 cohort at 1, 3, and 5 years were 0.661, 0.582, and 0.562, respectively (Fig. 2G).

### 3.3 Correlation between risk model and clinical features

**Fig. 3:**
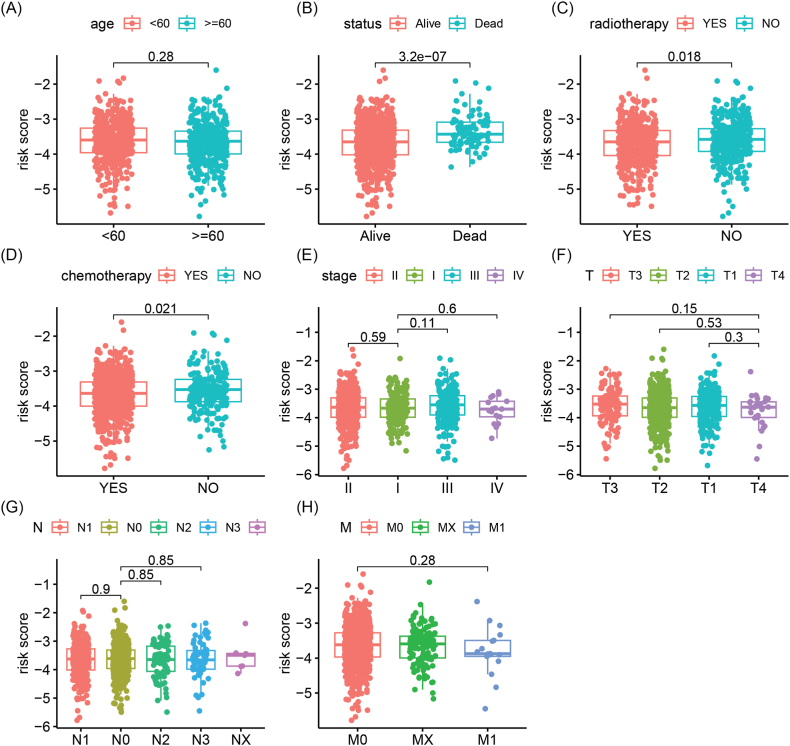
Comparison of risk scores of patients in different clinical and pathological groups. (A) Age (B) Survival outcome (C) Radiotherapy (D) Chemotherapy (E) Clinical stage (F) T stage (G) N stage (H) M stage.

We conducted an evaluation of the differences in risk scores among different clinical and pathological feature groups (Fig. 3A–H). The results indicated that the risk score of deceased patients was significantly higher than that of surviving patients (p = 3.2e-07), and the risk score of patients who received radiotherapy or chemotherapy was significantly lower than that of patients who did not receive radiotherapy (p = 0.018) or chemotherapy (p = 0.021). However, no significant differences in risk scores were found among patients of different ages, clinical stages, or TNM stages.

### 3.4 Gene expression characteristics of high-risk and low-risk patients

**Fig. 4:**
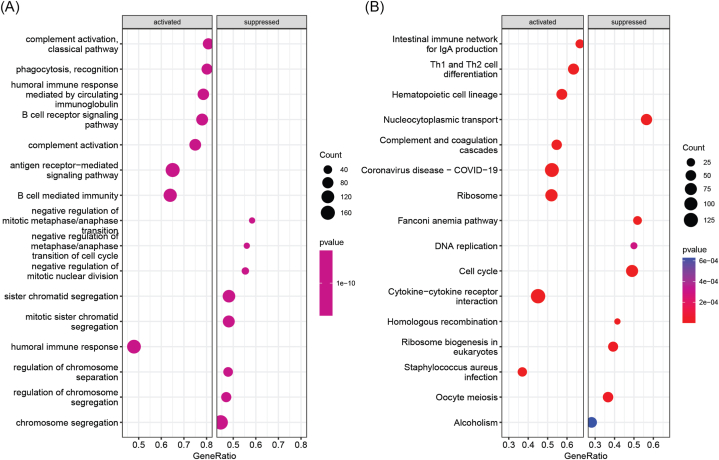
Gene set enrichment analysis between high-risk and low-risk groups. (A) Significantly activated and inhibited GO terms. (B) Significantly activated and inhibited KEGG pathways.

To investigate the differences in gene expression between high-risk and low-risk groups, we conducted gene set enrichment analysis. The results revealed that immune response-related processes were significantly activated in low-risk group patients, including humoral immune response, B cell mediated immunity, and antigen receptor-mediated signaling pathway. In contrast, chromosome-related processes were significantly suppressed, including chromosome segregation and sister chromatid segregation (Fig. 4A). Additionally, pathways such as cytokine-cytokine receptor interaction, ribosome, Th1 and Th2 cell differentiation were significantly activated, while pathways such as cell cycle and nucleocytoplasmic transport were significantly suppressed in the low-risk group (Fig. 4B).

### 3.5 Differences in immune infiltration between high-risk and low-risk patients

**Fig. 5:**
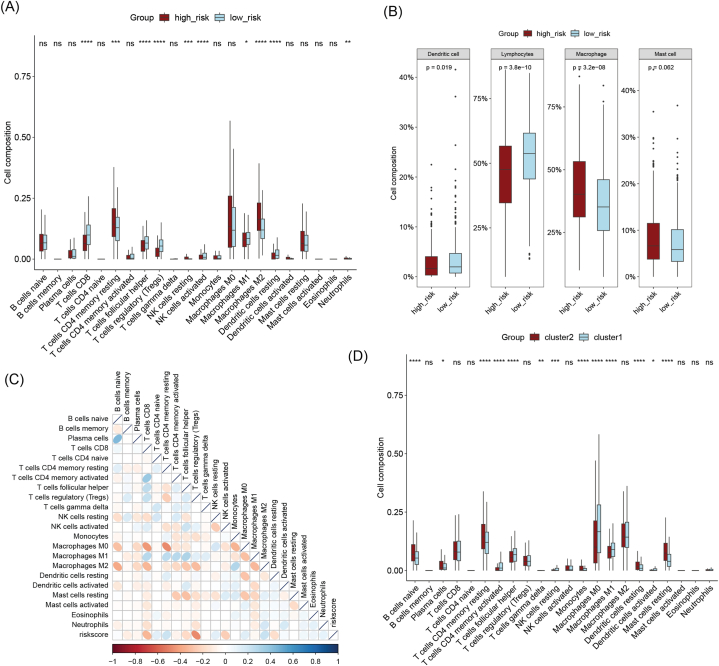
Relationship between risk features derived from lysosome-related genes and immune infiltration. (A) Comparison of immune cell infiltration in high-risk and low-risk group patients. (B) Comparative analysis of infiltration of four major immune cells in high-risk and low-risk group patients. (C) Correlation analysis between risk score and infiltration of 22 immune cells. (D) Comparative analysis of immune cell infiltration between molecular subtypes derived from lysosome-related genes. *p < 0.05,**p < 0.01,***p < 0.001,****p < 0.0001.

The degree and type of immune cell infiltration in tumor tissue are of significant value for tumor development and prognosis. Analysis indicates that there are notable differences in immune cell infiltration between high-risk and low-risk groups. In the high-risk group, the infiltration levels of CD8 T cells, follicular helper T cells, Tregs, activated NK cells, and resting dendritic cells were significantly higher than those in the low-risk group, while the infiltration levels of resting CD4 memory T cells and M2 macrophages were significantly lower than those in the low-risk group (Fig. 5A). Further classification revealed that macrophages were significantly increased in the high-risk group, while dendritic cells and lymphocytes were significantly decreased (Fig. 5B). Correlation analysis showed that the risk score was significantly negatively correlated with CD8 T cells and Tregs, while it was significantly positively correlated with resting CD4 memory T cells and M2 macrophages (Fig. 5C). Additionally, we observed that the immune infiltration between different subtypes had some similar trends with the immune infiltration in different risk groups, but there were slight differences in the infiltration of naive B cells and CD8 T cells (Fig. 5D). Cluster 1, which had a better prognosis, had higher B cell infiltration, while there was no significant difference in CD8 T cell infiltration among different molecular subtypes.

### 3.6 Differences in drug sensitivity between high-risk and low-risk patients

**Fig. 6:**
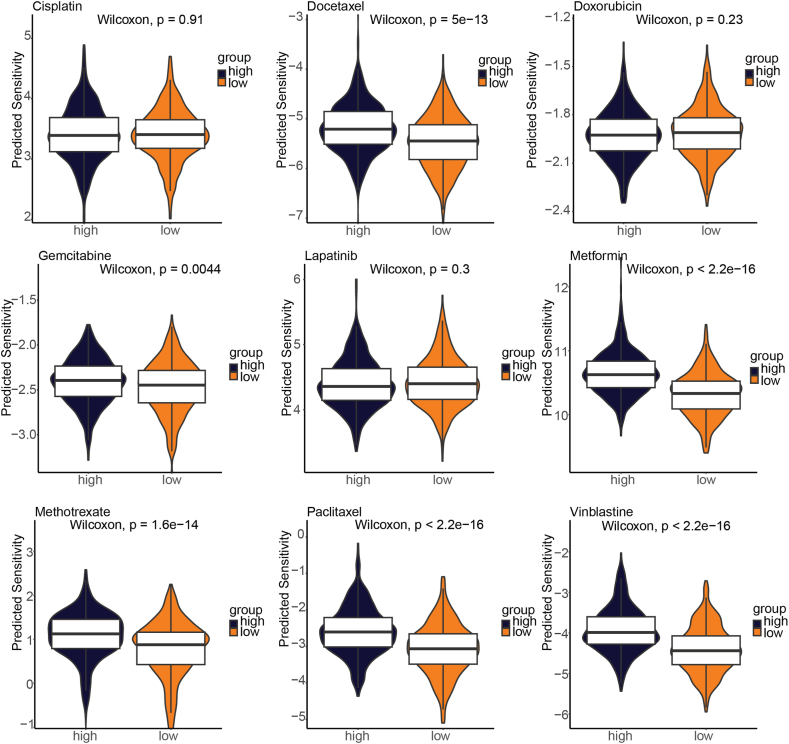
Comparative analysis of chemotherapy drug sensitivity between high-risk and low-risk group patients derived from lysosome-related genes.

We conducted an analysis on the TCGA-BRCA cohort to assess the sensitivity of 9 drugs and compared the differences in sensitivity between high-risk and low-risk groups (as shown in Fig. 6). Our findings indicate that patients in the low-risk group had lower sensitivity to docetaxel (p = 5e-13), gemcitabine (p = 0.0044), metformin (p < 2.2e-16), methotrexate (p = 1.6e-14), paclitaxel (p < 2.2e-16), and vinblastine (p < 2.2e-16) compared to those in the high-risk group. However, there was no significant difference in sensitivity to cisplatin, doxorubicin, and lapatinib between the two groups. These results suggest that patients in the low-risk group may benefit more from chemotherapy.

### 3.7 Differences in immune therapy response between high-risk and low-risk patients

**Fig. 7:**
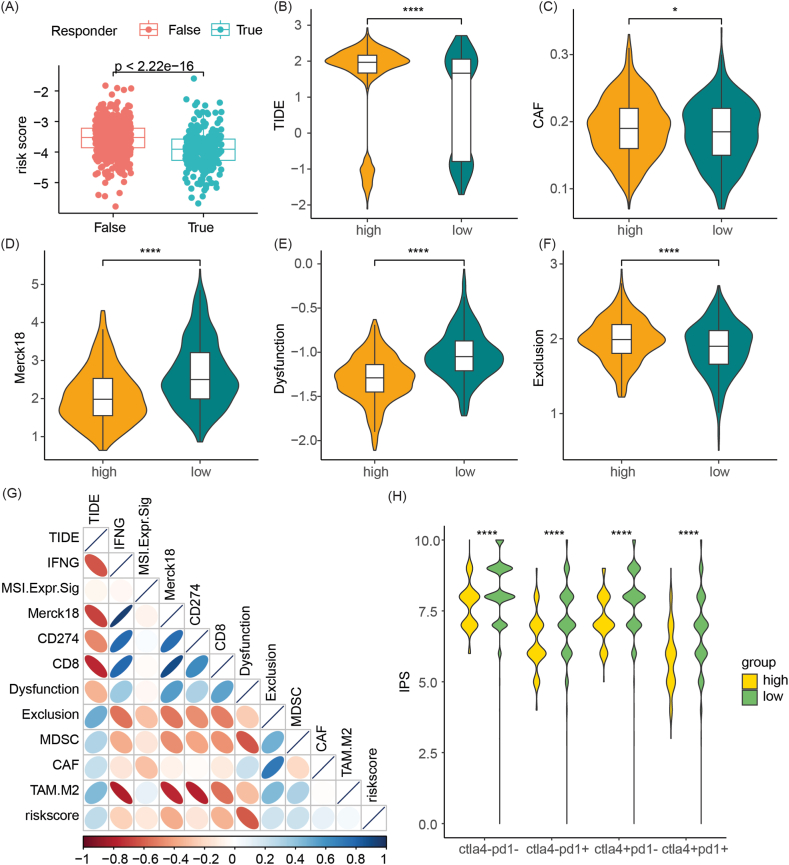
Comparative analysis of immune therapy response between high-risk and low-risk group patients derived from lysosome-related genes. (A) Comparison of risk scores between true responders and false responders. (B–F) Comparative analysis of TIDE, CAF, Merck18, Dysfunction, and Exclusion scores between high-risk and low-risk group patients. (G) Correlation analysis between risk score and immune therapy response-related predictive indicators. (H) Comparative analysis of IPS scores between high-risk and low-risk group patients. *p < 0.05,**p < 0.01,***p < 0.001,****p < 0.0001.

To further investigate, we assessed the response of patients in various risk categories to immunotherapy. Our findings indicated that the risk score of true responders was significantly lower than that of false responders (Fig. 7A). Furthermore, high-risk group patients exhibited higher TIDE, CAF, and Exclusion scores, as well as lower Merck18 and Dysfunction scores, compared to low-risk group patients (Fig. 7B–F). Correlation analysis revealed that the risk score was positively correlated with TIDE, CAF, TAM.M2, MDSC, and Exclusion, while negatively correlated with Merck18, Dysfunction, IFNG, CD8, among others (as illustrated in Fig. 7G). Additionally, regardless of CTLA and PD1 status, the IPS of high-risk group patients was significantly lower than that of low-risk group patients (Fig. 7H).

### 3.8 Independent prognostic factors and nomogram for breast cancer

**Fig. 8:**
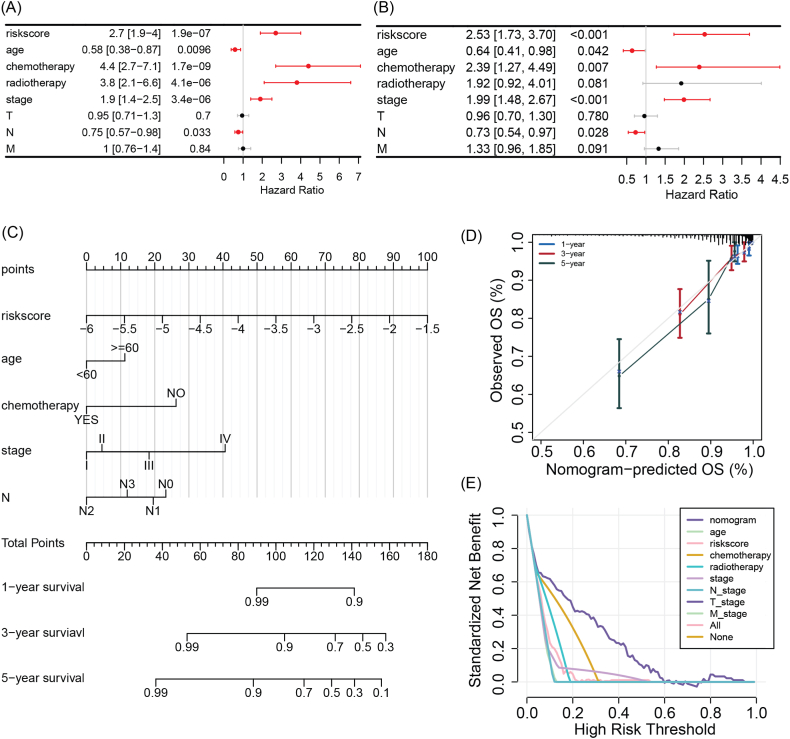
Independent prognostic factor analysis and nomogram construction and evaluation of breast cancer. (A) Univariate Cox regression analysis of riskscore and clinical pathological features. (B) Multivariate Cox regression analysis identifies riskscore, age, chemotherapy, stage, and N stage as independent prognostic factors for breast cancer. (C) Construction of a nomogram composed of riskscore, age, chemotherapy, stage, and N stage to predict 1, 3, and 5-year overall survival of breast cancer. (D) Calibration curve and (E) decision curve of the nomogram model.

A univariate Cox regression analysis was conducted, which revealed that riskscore, age, chemotherapy, radiotherapy, stage, and N stage were significantly associated with breast cancer prognosis (Fig. 8A). Additionally, a multivariate Cox regression analysis was performed, which identified riskscore, age, chemotherapy, stage, and N stage as independent prognostic factors for breast cancer (Fig. 8B). Consequently, a nomogram was constructed, consisting of these 5 independent prognostic factors, to predict the 1, 3, 5-year overall survival of breast cancer (Fig. 8C). The results indicated that the nomogram had good consistency with the actual observed overall survival, predicting 1, 3, and 5-year overall survival (Fig. 8D). Furthermore, compared with other individual prognostic factors such as age and staging, the entire independent prognostic factor nomogram had a better net benefit in prognostic prediction (Fig. 8E).

## 4 Discussion

Lysosomes are membrane-bound organelles that contain enzymes capable of breaking down various biomolecules, including proteins, lipids, and carbohydrates. They play an important role in cellular homeostasis and are involved in a variety of cellular processes, including autophagy [8], endocytosis [14], and phagocytosis [15]. Recent studies have suggested that lysosomes may also play a role in cancer development and progression. For example, lysosomal enzymes have been shown to promote tumor invasion and metastasis by degrading the extracellular matrix and promoting angiogenesis [16,17]. In addition, lysosomal dysfunction has been implicated in the development of various types of cancer, such as cervical cancer [18] and pancreatic cancer [19]. Targeting lysosomes may therefore represent a promising strategy for the development of new cancer therapies [20]. However, further research is needed to fully understand the role of lysosomes in cancer and to develop effective lysosome-targeted therapies.

In this study, 176 lysosome-related genes associated with breast cancer prognosis were screened, and two molecular subtypes of breast cancer were identified based on the transcriptional profiles of these genes. These subtypes showed significant differences in prognosis and immune infiltration. Additionally, a new breast cancer prognostic model consisting of 7 lysosome-related genes was constructed, which has potential for prognostic prediction, evaluation of immune therapy response, and drug sensitivity. Furthermore, independent prognostic factors for breast cancer were identified, including age, chemotherapy, clinical stage, N stage, and risk models derived from lysosome-related genes. A nomogram suitable for clinical use was constructed, which performed well in predicting overall survival and deserves further clinical validation and promotion.

The prognostic signature in this study is composed of seven genes, namely BBC3, GDI2, HLA-DQB2, HSP90AA1, HSPA8, LAMTOR4, and PSMD1. BBC3 is a pro-apoptotic gene that has been associated with breast cancer phenotype and prognosis through the rs2032809 polymorphism [21]. GDI2 is highly expressed and closely associated with the occurrence and development of many tumors, making it a potential diagnostic and prognostic biomarker for hepatocellular carcinoma [22]. Additionally, GDI2 is a target of paclitaxel through the p75NTR signaling pathway in prostate cancer tumorigenesis [23]. HLA-DQB2 has been shown to be highly expressed in breast cancer and predicts better overall survival [24]. Plasma HSP90AA1 has been found to predict the risk of breast cancer incidence and distant metastasis [25]. It is worth noting that HSP90AA1 has been associated with cancer drug resistance in multiple studies, including osteosarcoma [26] and ovarian cancer [27]. Recent studies have shown that HSPA8 can inhibit ferroptosis to support liver cancer progression [28]. HSPA8 expression is related to TNM staging of triple-negative breast cancer and has important prognostic value [29]. Additionally, targeting HSPA8 by downregulating BCR-ABL can enhance the chemotherapy sensitivity of chronic myeloid leukemia cells [30]. The lysosomal TMEM9-LAMTOR axis has been found to play an important role in controlling mTOR signaling integrity in breast tumor development [31]. PSMD1 is a 26S proteasome subunit that regulates breast cancer cell growth by degrading p53 protein [32]. Cell experiments have shown that reducing the 26S/20S ratio may be a valuable strategy for treating drug-resistant invasive cancers [33].

Tumor tissues can infiltrate various types of immune cells, including T cells, B cells, natural killer cells, macrophages, and dendritic cells. Our analysis found that there is lower infiltration of resting CD4 memory T cells in subtypes with better prognosis or low-risk groups, while the infiltration levels of follicular helper T cells and M1-type macrophages are increased. This suggests that the infiltration of these immune cells may be related to tumor development and prognosis. Resting CD4 memory T cells are characterized by their ability to persist in the body for a long time without antigen stimulation and their ability to respond quickly to antigen re-exposure, making them an important component of the immune system's memory response [34]. Follicular helper T cells are characterized by the expression of transcription factor Bcl6 and surface markers CXCR5 and PD-1. These cells participate in various immune functions, including regulating B cell differentiation and producing cytokines such as IL-21 and IL-4. The presence of Tfh cells often coincides with better prognosis. Studies have shown that CD4^+^ follicular helper T cell infiltration predicts breast cancer survival [35]. Recent studies have shown that follicular helper T cells can restore or promote CD8^+^ dependent anti-tumor immunity [36,37]. Tumor-associated macrophages (TAMs) play a crucial role in promoting cancer progression. M2-type macrophages have anti-inflammatory and immune-suppressive characteristics, while classically activated M1-like macrophages have been shown to have pro-inflammatory and anti-tumor immune activity [38]. The immune infiltration characteristics that may result in immune suppression and immune activation states explain the differences in prognosis between different risk groups and molecular subtypes. Additionally, our findings indicate significant differences in immune therapy response among different patient groups, which may be related to the immune infiltration characteristics of the patients [39].

Drug resistance remains the main cause of chemotherapy failure and cancer-related mortality. Lysosomes play a crucial role in the formation of drug resistance as a tumor-resistant medium, and they also provide potential solutions to overcome drug resistance. Notably, there is a significant difference in drug sensitivity between high-risk and low-risk patients, particularly in their sensitivity to docetaxel, gemcitabine, metformin, methotrexate, paclitaxel, and vinblastine. This may be related to the genes that affect drug sensitivity, such as GDI2, HSP90AA1, and HSPA8. However, the relationship between these genes and other undisclosed genes related to chemotherapy sensitivity has not been fully elucidated in the mechanism of drug resistance in breast cancer, which warrants further exploration in subsequent research to provide more targets for solving drug resistance problems. There are also some limitations to this study. The data used in this study are all from the TCGA and GEO databases, so the constructed prognostic model needs to be validated with more extensive clinical data. Additionally, the biological functions of some key genes are cited through historical literature, and more in vitro and in vivo studies are needed to elucidate them.

## 5 Conclusion

To summarize, this research has identified two molecular subtypes of breast cancer that exhibit significant differences in prognosis and immune infiltration based on LRGs. A novel prognostic feature derived from LRGs has been developed, and a nomogram model for clinical prognosis analysis of breast cancer has been constructed by combining the signature, age, chemotherapy, clinical stage, and N stage. This study provides novel insights into the mechanisms of LRGs in the onset and progression of breast cancer, and presents potential targets for drug development and addressing drug resistance in breast cancer.

## Funding statement

This study was supported by 10.13039/501100017594Medical and Health Science and Technology Project of Zhejiang Province (2023KY505; 2023KY046). Traditional Chinese Medicine Science and Technology Project of Zhejiang Province (2023ZL273).

## Data availability statement

The extracted data used to support the findings of this study are included within the article.

## CRediT authorship contribution statement

**Xiaozhen Liu:** Conceptualization, Data curation, Formal analysis, Methodology, Writing – original draft. **Kewang Sun:** Data curation, Formal analysis, Visualization. **Hongjian Yang:** Data curation, Formal analysis, Visualization. **Dehomg Zou:** Data curation, Formal analysis, Visualization. **Lingli Xia:** Data curation, Formal analysis, Visualization. **Kefeng Lu:** Data curation, Formal analysis, Visualization. **Xuli Meng:** Data curation, Formal analysis, Visualization, Writing – review & editing. **Yongfeng Li:** Conceptualization, Data curation, Formal analysis, Funding acquisition, Writing – original draft, Writing – review & editing.

## Declaration of competing interest

The authors declare that the research has no competing interests.

## Figures and Tables

**figs1:**